# Mushroom poisoning with cardiogenic shock caused by *Russula subnigricans* successfully treated with mechanical circulatory support devices: a case report

**DOI:** 10.1093/ehjcr/ytae337

**Published:** 2024-07-12

**Authors:** Shota Iseki, Shogo Yamaguchi, Yuta Ozaki, Yusuke Uemura

**Affiliations:** Cardiovascular Center, Anjo Kosei Hospital, Anjo, Aichi, 446-8602, Japan; Cardiovascular Center, Anjo Kosei Hospital, Anjo, Aichi, 446-8602, Japan; Cardiovascular Center, Anjo Kosei Hospital, Anjo, Aichi, 446-8602, Japan; Cardiovascular Center, Anjo Kosei Hospital, Anjo, Aichi, 446-8602, Japan

**Keywords:** Mushroom poisoning, *Russula subnigricans*, Cardiogenic shock, Mechanical circulatory support, Case report

## Abstract

**Background:**

Mushroom poisoning caused by the ingestion of *Russula subnigricans* has been reported to cause rhabdomyolysis and cardiac dysfunction, leading to death. There have been few reports of cardiogenic shock induced by mushroom poisoning that was successfully treated using mechanical circulatory support devices.

**Case Summary:**

A 38-year-old man presented with gastrointestinal symptoms a day after consuming a curry made with forest-collected mushrooms and was admitted with a diagnosis of rhabdomyolysis. Despite appropriate fluid management for severe rhabdomyolysis, the patient experienced anuria and cardiogenic shock with a remarkably reduced left ventricular ejection function, followed by the development of ventricular fibrillation. Mechanical support using Impella CP, veno-arterial extracorporeal membranous oxygenation (VA-ECMO), and continuous haemodiafiltration were administered for cardiogenic shock and severe rhabdomyolysis. His cardiac and renal function gradually improved, and the patient was successfully weaned off VA-ECMO on day 4, Impella CP on day 5, and renal replacement therapy on day 23. The left ventricular ejection fraction returned to normal without any neurological, respiratory, or renal sequelae. The remaining mushroom samples were identified as *R. subnigricans* by polymerase chain reaction testing.

**Discussion:**

This is the first reported case of cardiogenic shock caused by *R. subnigricans* poisoning, successfully treated with Impella CP and VA-ECMO. The optimal use of mechanical circulatory support devices plays an important role in the treatment of cardiogenic shock caused by mushroom toxicity.

**Learning points**

- Mushroom poisoning by *Russula subnigricans* could induce cardiogenic shock.
- Optimal implementation of mechanical circulatory support can stabilise the haemodynamics.
- Cardiac dysfunction caused by the toxicity of *R. subnigricans* is reversible.

## Introduction

*Russula subnigricans* is a poisonous mushroom, and poisoning due to mistaken ingestion occurs mainly in East Asia.^1,2^ The typical symptoms of poisoning include gastrointestinal symptoms, including vomiting and diarrhoea, which appear approximately 30 min after ingestion, followed by weakness and myalgia. In severe cases, patients develop rhabdomyolysis, accompanied by acute renal failure and electrolyte disturbances, finally leading to death.^2,3^ Furthermore, cardiac disturbances including abnormal changes in electrocardiograms and systolic dysfunction, ventricular arrhythmia, and haemodynamic collapse have also been documented.^1,3,4^ The mortality rate due to *R. Subnigricans* mushroom poisoning has been reported to be about 50%.^5,6^ Due to the fulminant progression and high fatality rate, the mechanisms of severe cardiac dysfunction have not been fully elucidated. Herein, we report a case of mushroom poisoning caused by *R. Subnigricans* with severe rhabdomyolysis and cardiogenic shock that was successfully treated using mechanical circulatory support devices, along with insights from endomyocardial biopsy and cardiac magnetic resonance imaging (MRI).

## Summary figure

**Table:** 

Two days before transfer to our hospital	The patient ingested a curry made with forest-collected mushrooms.
The day before transfer	The patient presented with diarrhoea, vomiting, and systemic muscle pain and was admitted to a local hospital with a diagnosis of rhabdomyolysis
Day 0 (Admission to our hospital)	The patient was transferred to our hospital because of cardiogenic shock with severely reduced left ventricular ejection fraction. Mechanical support with an Impella CP was initiated
Three hours after admission	Owing to haemodynamic collapse caused by ventricular fibrillation, veno-arterial extracorporeal membranous oxygenation (VA-ECMO) was performed
Twelve hours after admission	Continuous haemodiafiltration was initiated due to pulmonary oedema and anuria
Day 4	The patient was weaned off VA-ECMO
Day 5	The patient was weaned off Impella CP
Day 23	The patient stopped renal replacement therapy
Day 41	Discharge to home without neurological, cardiovascular, respiratory, or renal sequelae

## Case presentation

A 38-year-old man with no medical history and no regular medications presented to a nearby hospital with diarrhoea, vomiting, and systemic muscle pain 1 day after eating a curry made with forest-collected mushrooms. He was admitted with rhabdomyolysis, which was suspected to be caused by mushroom poisoning. Despite fluid replacement management, he developed anuria and cardiogenic shock with remarkably reduced left ventricular ejection function and was referred to our hospital 2 days after the ingestion.

**Figure 1:**
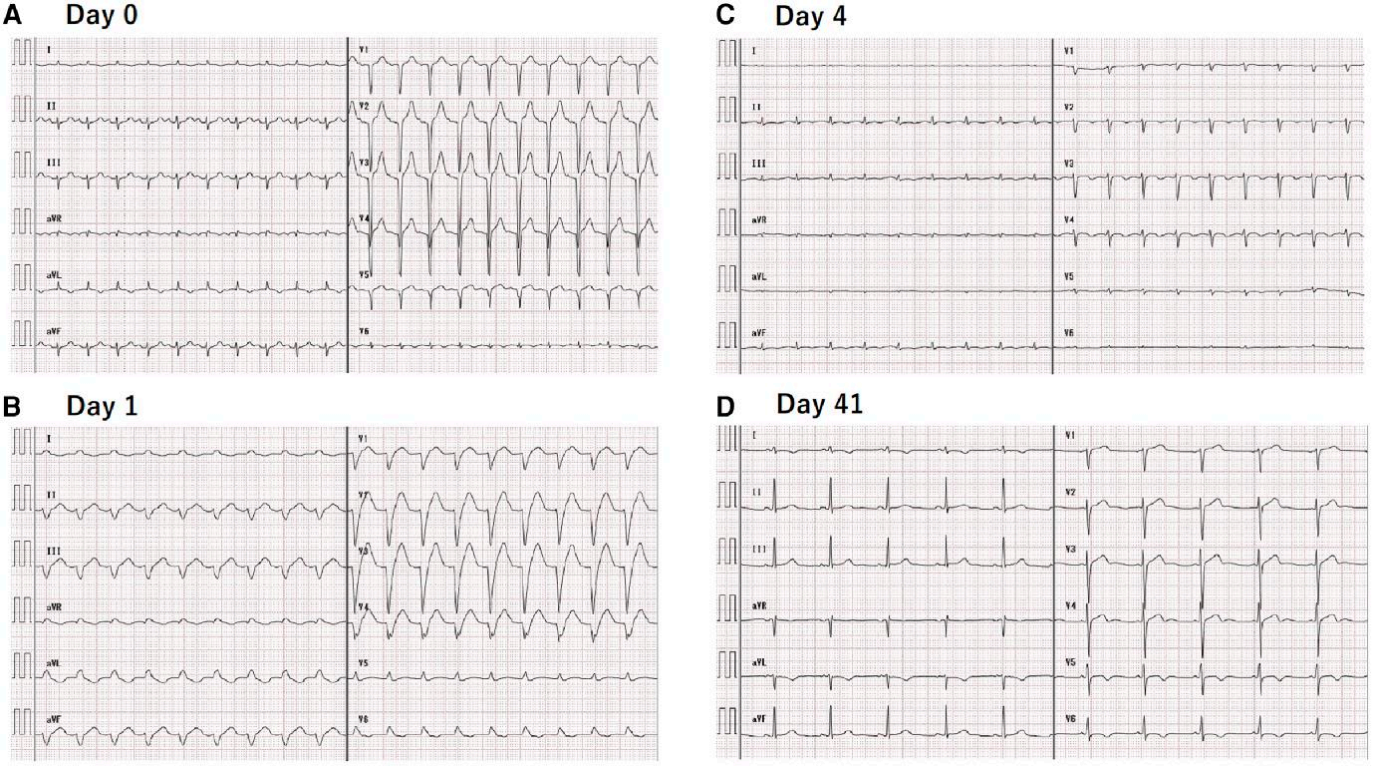
Serial changes in 12-lead electrocardiograms. (*A*) Sinus rhythm, left axis deviation, poor R wave progression in the precordial leads, and negative T waves in leads I and aVL at admission. (*B*) Left bundle branch block and left axis deviation with unknown rhythm on day 1. (*C*) Low voltage in the precordial leads with the recovery to the narrow QRS complex and sinus rhythm on day 4. (*D*) Sinus rhythm with the normal voltage and R wave progression in the precordial leads only with negative T waves in the I, aVL, V5, and V6 leads on day 41.

On presentation, he was intubated due to respiratory failure and was in shock with sweating and cold extremities, a heart rate of 126/min and blood pressure of 82/60 mmHg (dobutamine 2 µg/kg/min). During the initial examination at our hospital, serum creatinine kinase (CK) levels were remarkably elevated (185 928 IU/L; reference, 41–153 IU/L). His white blood cell count (12.5 × 10^3^ cells/μL; reference, 3.3 × 10^3^–8.6 × 10^3^ cells/μL), serum lactate level (4.2 mmol/L; reference, 0.5–2.0 mmol/L), cardiac troponin T level (1.270 ng/mL; reference, 0–0.100 ng/mL), and N-terminal pro-B-type natriuretic peptide level (16 439 pg/mL; reference, 0–125 pg/mL) were also elevated. CK isozyme levels were measured, and CK-MB was approximately 6%. Electrocardiography at admission showed sinus rhythm, left axis deviation, poor R wave progression in the precordial leads, and negative T waves in leads I and aVL (*Figure 1A*). Transthoracic echocardiography performed in the emergency department revealed a severely reduced left ventricular ejection fraction of 20%.

To stabilise the haemodynamics, he was promptly moved to the catheterisation room, and mechanical circulatory support using Impella CP (Abiomed, Inc., Danvers, MA, USA), a percutaneous transvalvular microaxial flow pump, was initiated. The results of right heart catheterisation after Impella CP insertion were as follows: right atrial pressure, 8 mmHg; pulmonary artery pressure, 25/13 mmHg; and pulmonary artery wedge pressure, 15 mmHg. The cardiac index was 2.3 L/min/m^2^, and venous oxygen saturation was 78% on P9 support. During the subsequent endomyocardial biopsy, the mean blood pressure was maintained at >65 mmHg, and lactate levels gradually decreased. However, approximately 3 h after admission to the intensive care unit, electrocardiography demonstrated ventricular tachycardia. Impella support was maintained at P9 without a suction alarm. Despite our attempts to suppress ventricular tachycardia by the position change of Impella CP, sedation control, and the administration of amiodarone, the patient deteriorated into incessant ventricular fibrillation. Therefore, we decided to implement cardiopulmonary support with veno-arterial extracorporeal membrane oxygenation (VA-ECMO). Due to pulmonary oedema and anuria, continuous haemodiafiltration was initiated 12 h after admission.

The cardiac and renal function gradually improved, and the patient was successfully weaned off VA-ECMO on day 4, Impella CP on day 5, and renal replacement therapy on day 23 after admission. Electrocardiograms showed dramatic changes during hospitalisation along with the patient’s medical condition (*Figures 1A–D*). After rehabilitation, the patient was discharged on day 41 without any neurological, cardiovascular, respiratory, or renal sequelae.

**Figure 2:**
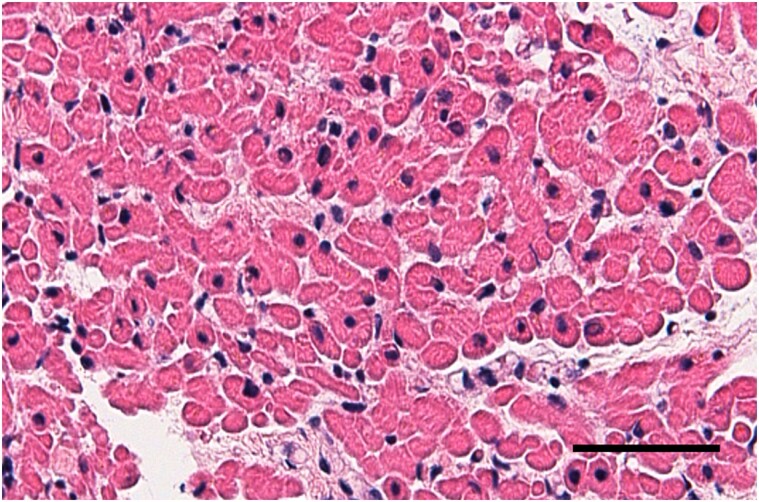
Endomyocardial biopsy on admission. Haematoxylin and eosin staining shows no obvious findings of inflammatory cell infiltration or myocyte destruction. Black bar indicates 50 µm.

**Figure 3:**
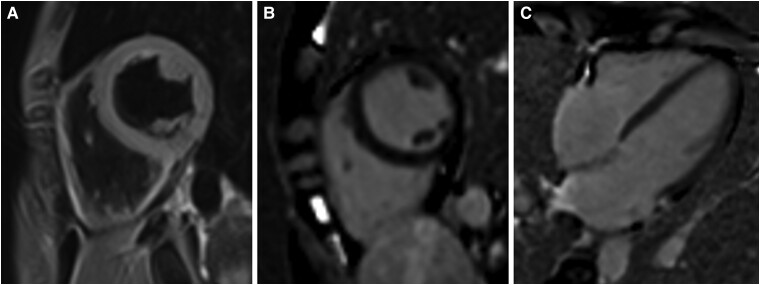
T2-weighted and gadolinium-enhanced cardiac magnetic resonance imaging. (*A*) Short axis view of T2-weighted short-tau inversion recovery imaging. Regional high signal intensities or increased global signal intensity ratio was not identified in the myocardium. (*B*) Short axis and (*C*) Four-chamber view of gadolinium-enhanced imaging. Delayed contrast was not identified in the myocardium.

**Figure 4:**
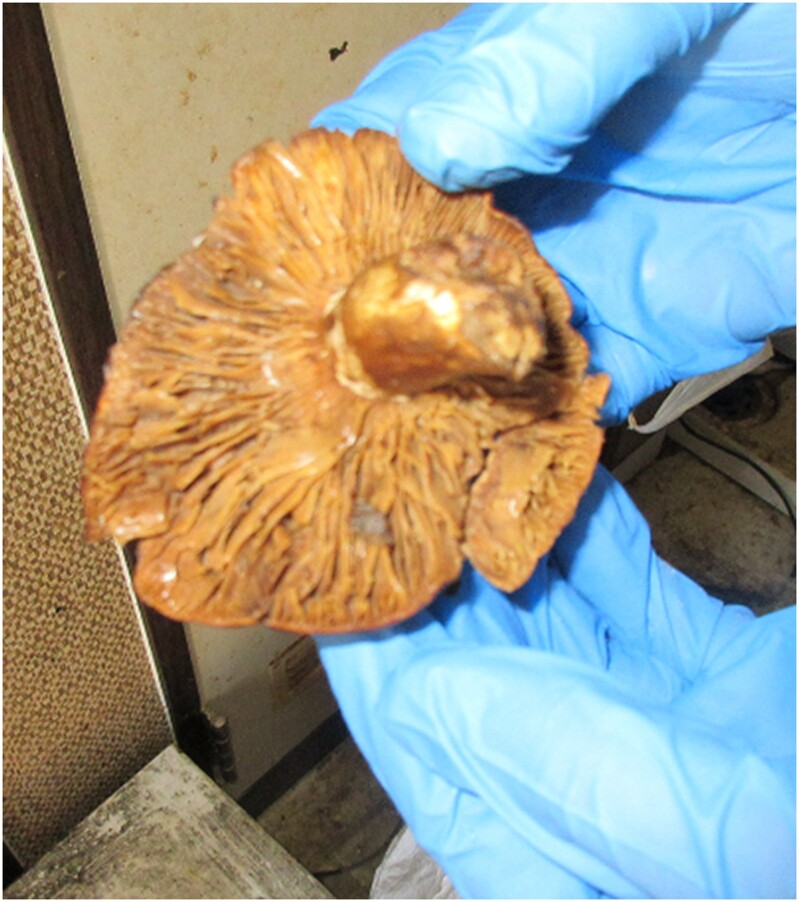
A leftover sample of the mushroom responsible for our case.

Inflammatory cell infiltration and myocyte destruction were not identified in the right ventricular endomyocardial biopsy on admission (*Figure 2*). Furthermore, gadolinium-enhanced cardiac MRI performed on day 47 revealed no findings of T2-weighted and late gadolinium enhancement imaging in the myocardium (*Figure 3*). The mushrooms were identified as *R. subnigricans* by the analysis of toxin indicators and polymerase chain reaction gene searches of the leftover samples (*Figure 4*).

## Discussion

The present case of mushroom poisoning by *R. subnigricans,* which manifested as severe rhabdomyolysis and cardiogenic shock, was successfully treated with Impella CP and VA-ECMO.

Clinical manifestations of mushroom poisoning depend on the toxin of the mushroom species consumed. General management of mushroom poisoning mainly consists of supportive therapy, such as fluid resuscitation and antiemetics for gastrointestinal symptoms. The administration of activated charcoal or specific antidote is also a therapeutic option. In some cases, delayed- or late-onset syndromes could be life-threatening due to liver and renal failure, requiring intensive care. Therefore, clinical diagnosis and evaluation of mushroom poisoning, careful observation, and proper management for the lethal manifestations are required.^7,8^

*R. subnigricans* poisoning was first reported in Japan in 1954, and seven outbreaks (15 poisonings and seven deaths) were reported in 2007.^6^ Cycloprop-2-ene carboxylic acid is a toxic component of *R. subnigricans,* which causes rhabdomyolysis in mice.^9^ However, the mechanisms underlying cardiac disturbances in patients with mushroom poisoning remain unknown. Previous reports on patients with cardiac dysfunction due to mushroom poisoning did not fully examine their pathophysiology.^1,3,4^ In the present case, the cardiac function fully recovered, with no pathological inflammation in the endomyocardial biopsy and no evidence of myocardial damage on cardiac MRI. This indicates that *R. subnigricans* poisoning has an indirect influence on cardiac function, probably due to free radicals, cytokines, or haemodynamic stress.

Considering that the cardiac dysfunction caused by *R*. *subnigricans* was reversible, mechanical circulatory support seems to be crucial for haemodynamic stabilisation as a bridge to recovery. Based on the judgement of our patient’s status of Society for Cardiac Angiography and Interventions Shock Stage C, an Impella CP was inserted.^10^ There has been a case report of cardiogenic shock following *Amanita proxima* mushroom poisoning treated using an intra-aortic balloon pump.^11^ The Impella CP can provide more powerful support and unloading of the left ventricle compared with an intra-aortic balloon pump,^12^ and the additive implementation with VA-ECMO against haemodynamic collapse due to fatal arrhythmia contributed to the stabilisation of haemodynamics and successful recovery without any sequelae. This case indicates that the use of mechanical circulatory support could optimise haemodynamics and allow multimodal treatment, even in otherwise fatal cases of mushroom toxicity with cardiac dysfunction.

To the best of our knowledge, this is the first case of *R. subnigricans* poisoning with cardiogenic shock successfully treated with Impella CP and VA-ECMO. Given that cardiac dysfunction is indirectly caused and is reversible, the optimal use of mechanical circulatory support devices is important to achieve haemodynamic stability and increase the survival rate in the treatment of mushroom poisoning with cardiogenic shock.

## Data Availability

The data that support the findings of this study are available from the corresponding author upon reasonable request.

## Lead author biography

Shota Iseki is a resident physician of the Cardiovascular Center at the Anjo Kosei Hospital in Aichi, Japan. He received his MD degree from Shinshu University, Nagano, Japan, in 2019. His research interests are mainly in the field of interventional cardiology for cardiogenic shock.
